# Data showing non-conventional HLA-B27 expression in axial joints and gut tissue from B27 transgenic rats, and in frozen and paraffin-fixed synovial SpA tissue

**DOI:** 10.1016/j.dib.2016.08.046

**Published:** 2016-08-28

**Authors:** Oliwia Rysnik, Kirsty McHugh, Leonie van Duivenvoorde, Melissa van Tok, Joel Taurog, Simon Kollnberger, Dominique Baeten, Paul Bowness

**Affiliations:** a Nuffield Department of Orthopaedics, Rheumatology and Musculoskeletal Science, University of Oxford, Oxford, UK; b Amsterdam Rheumatology and Immunology Center, Department of Clinical Immunology and Rheumatology, Department of Experimental Immunology Academic Medical Center, University of Amsterdam, Amsterdam, The Netherlands; c Department of Internal Medicine, Rheumatic Diseases Division, University of Texas Southwestern Medical Center, Dallas, USA; d Cardiff Institute of Infection & Immunity, Henry Wellcome Building, Heath Park, Cardiff CF14 4XN, UK

**Keywords:** HLA class I free-heavy chains, HLA-B27, HLA-B27 transgenic rat model, Spondyloarthropathies

## Abstract

Data is presented showing expression of non-conventional (NC) heavy chain forms of B27 in synovial tissues from SpA patients. Data is presented showing the expression patterns of NC-B27 in joint, gastrointestinal and lymphoid tissues from B27 transgenic (TG^1^) rats with *M. tuberculosis*-induced SpA. Expression of NC-B27 was determined by immunohistochemistry and flow cytometry using HC10 and HD6 antibodies. These data are the extension of the data presented and discussed in “Non-conventional forms of HLA-B27 are expressed in Spondyloarthritis joints and gut tissue” (O. Rysnik, K. McHugh, L. M. van Duivenvoorde, M. N. van Tok, G. Guggino, J. D. Taurog, S. Kollnberger, F. Ciccia, D. L. Baeten, P. Bowness, 2016) [1].

**Specifications Table**

**Table:** 

Subject area	*Biology*
More specific subject area	*Human and rat spondyloarthritis*
Type of data	*Figures*
How data was acquired	*Histology -AperioCS2 Scanner (Leica Biosystem) Flow cytometry - BD FACS Canto*
Data format	*Analyzed*
Experimental factors	*Human and rat tissue*
Experimental features	*Antibody staining documented by histology and FACS*
Data source location	*Oxford UK*
Data accessibility	*Data is with this article*

**Value of the data**

• Data presented in this article confirm the role of NC-B27 in SpA pathogenesis in both human and transgenic rats.
• This data serves as a benchmark for future studies on the pathogenic role of NC-B27 in SpA.
• The data is valuable for future studies on development of novel treatment strategies for SpA.

## 1 Data

**Fig. 1:**
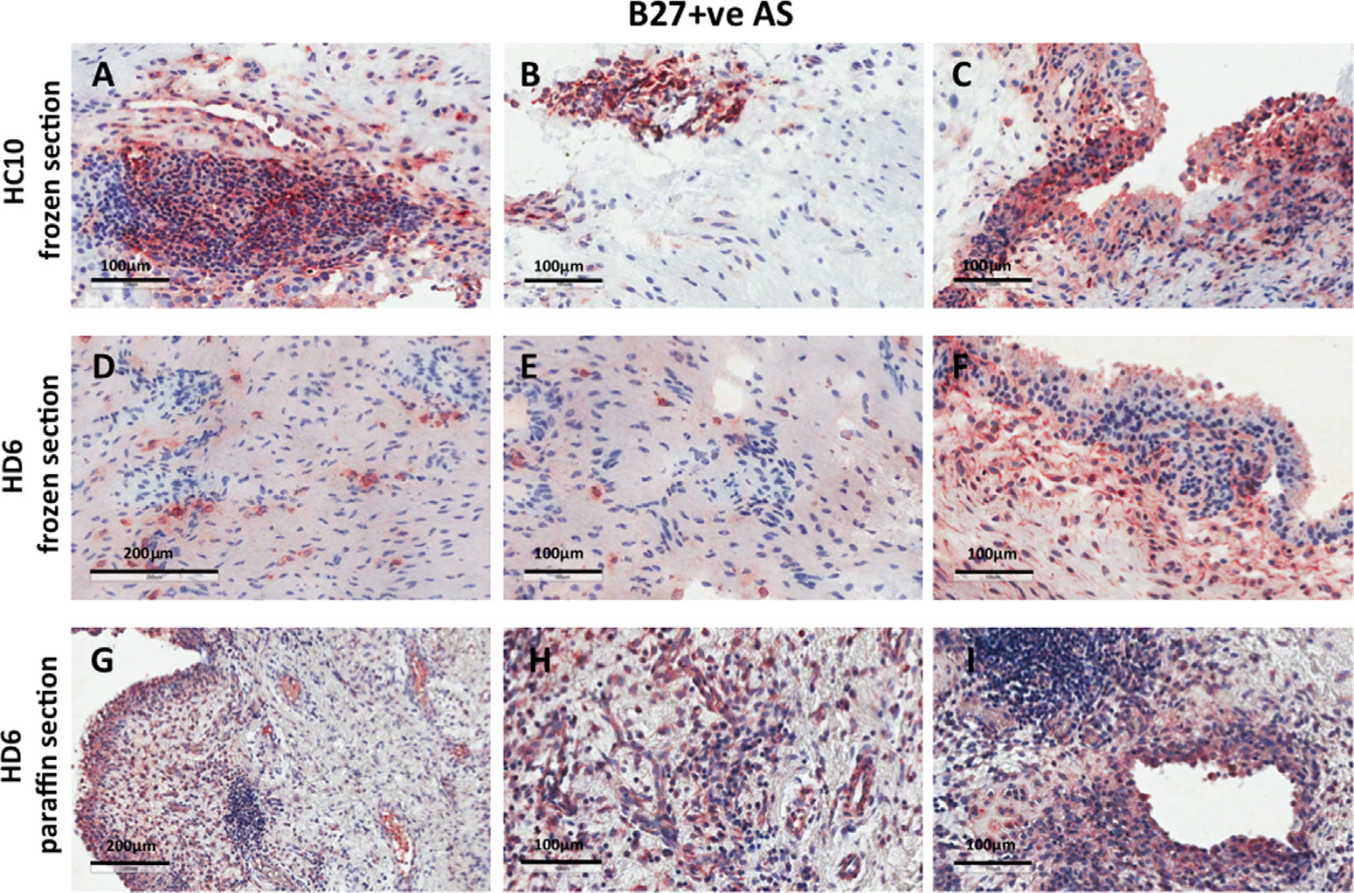
shows HC10 and HD6 staining of both frozen and paraffin-fixed synovial tissues from patients with HLA-B27-positive Spondyloarthritis (SpA).

**Fig. 2:**
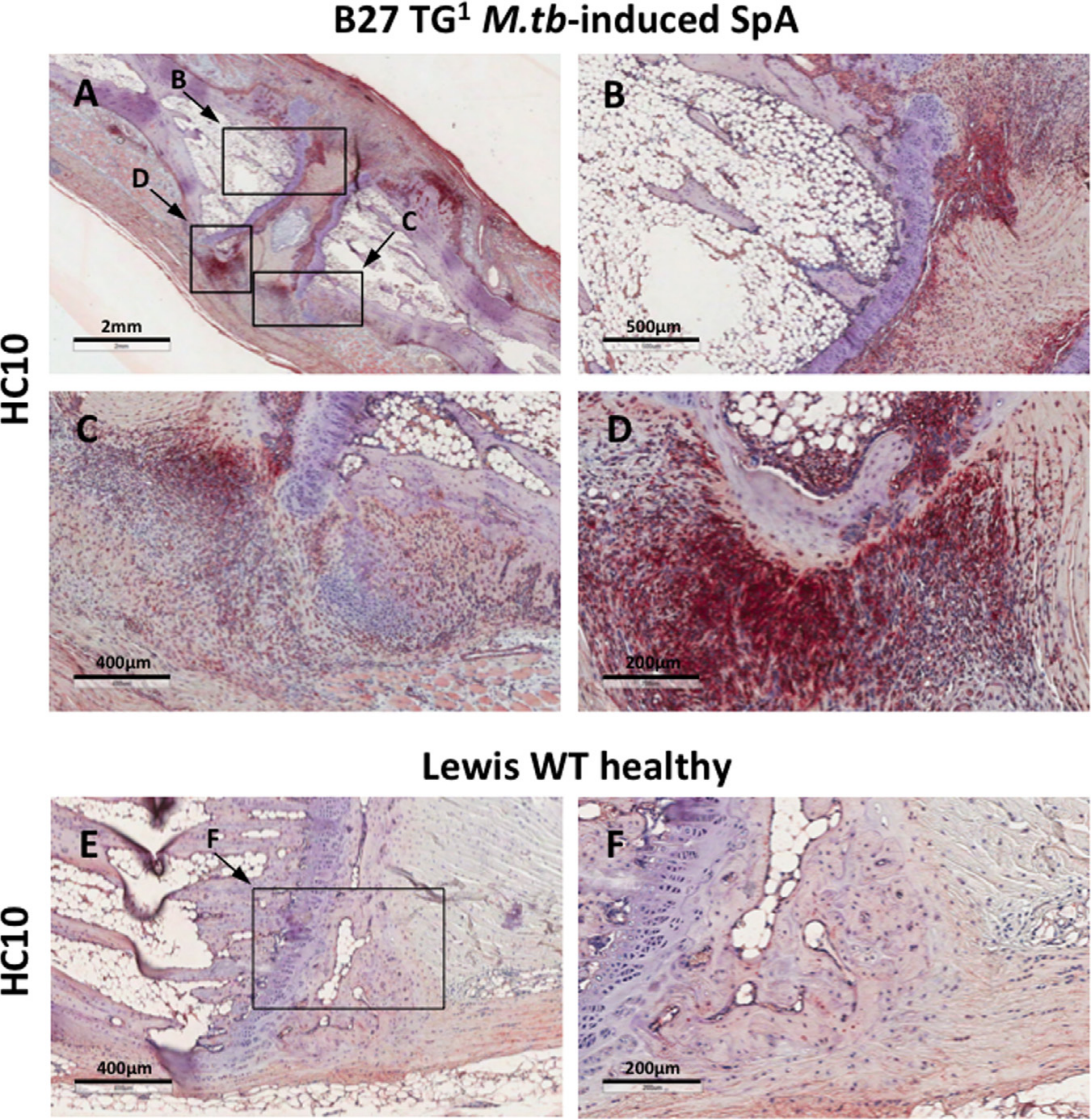
**(A–D)** shows HC10 staining of axial joints from B27 TG^1^ rats with *M.tb*-induced SpA. Staining was observed particularly in cell infiltrates at the junction between the vertebrae, connective tissue and annulus fibrosus. We did not observe HC10 staining in ankle or tail joints from healthy Lewis WT rats (Fig. 2**E and F)**.

**Fig. 3:**
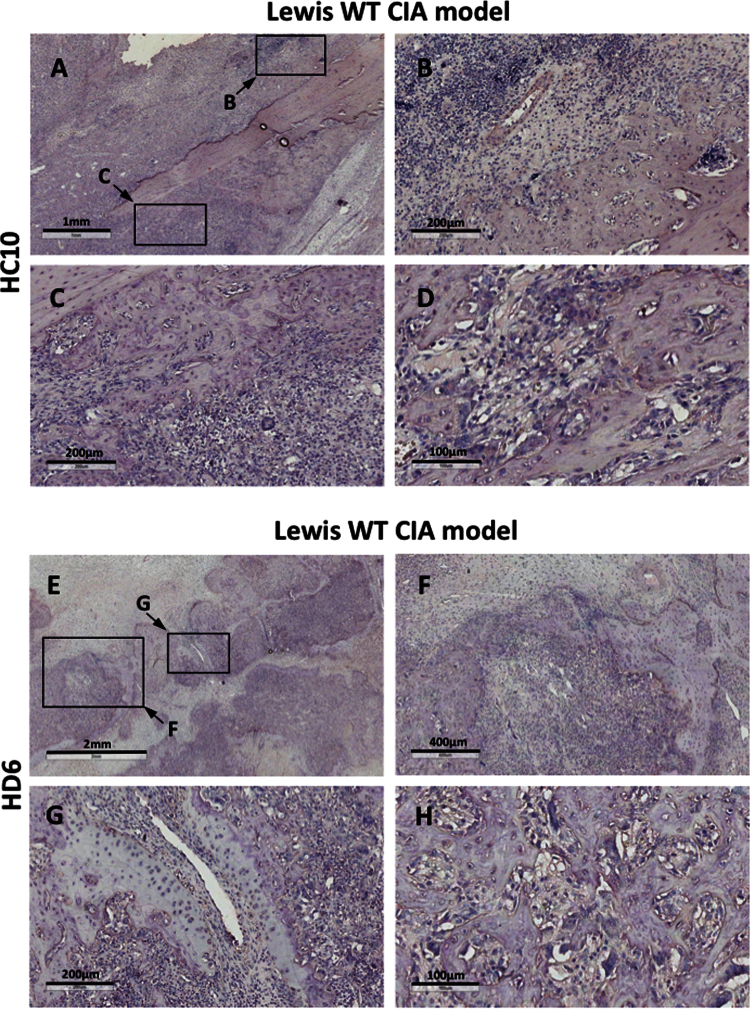
We did not observe HC10 or HD6 staining in ankle joints from Lewis WT rats with adjuvant-induced arthritis (AIA).

**Fig. 4:**
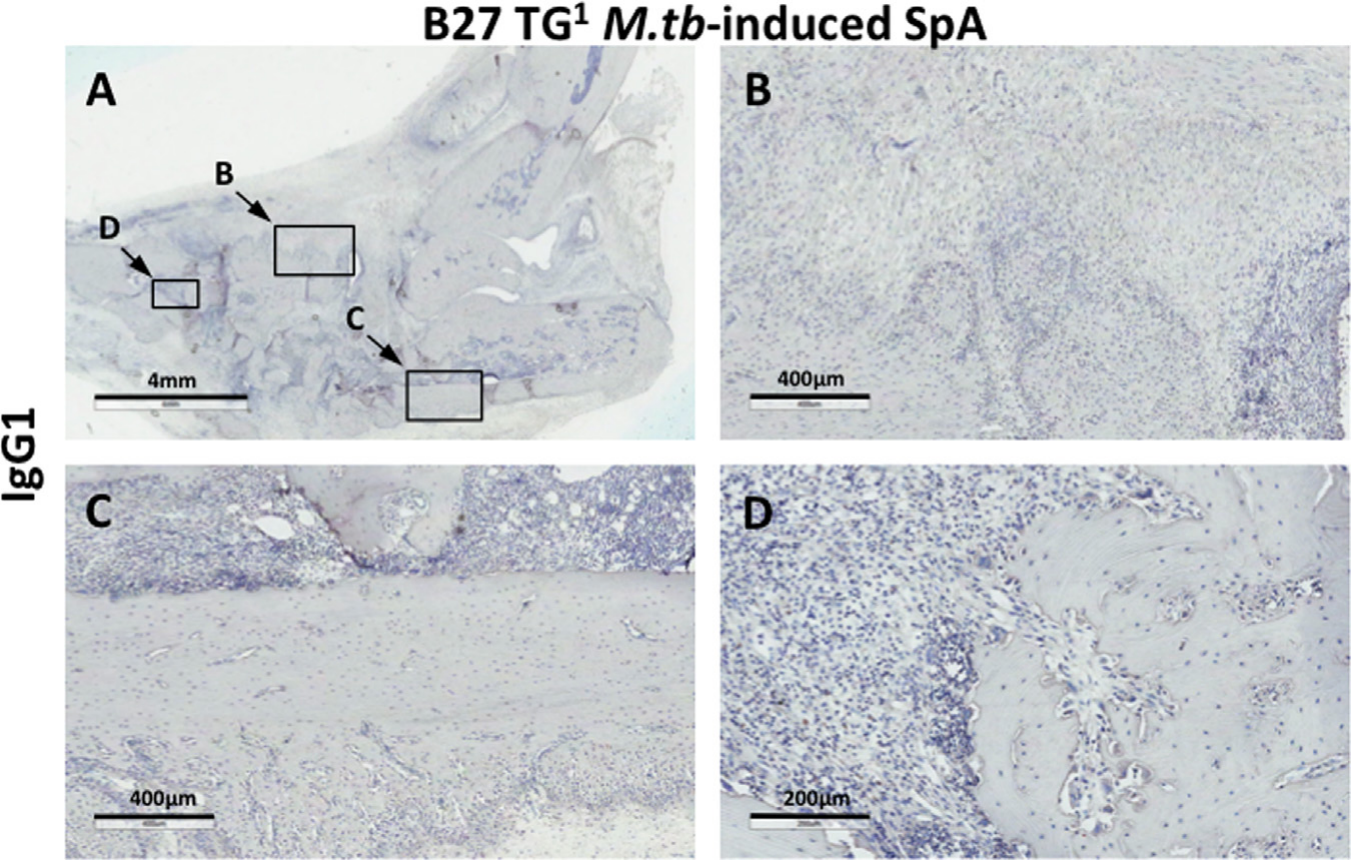
Shows that tissue sections from B27 TG^1^ rats with *M.tb*-induced arthritis and spondylitis did not stain with IgG1 isotype control antibody.

**Fig. 5:**
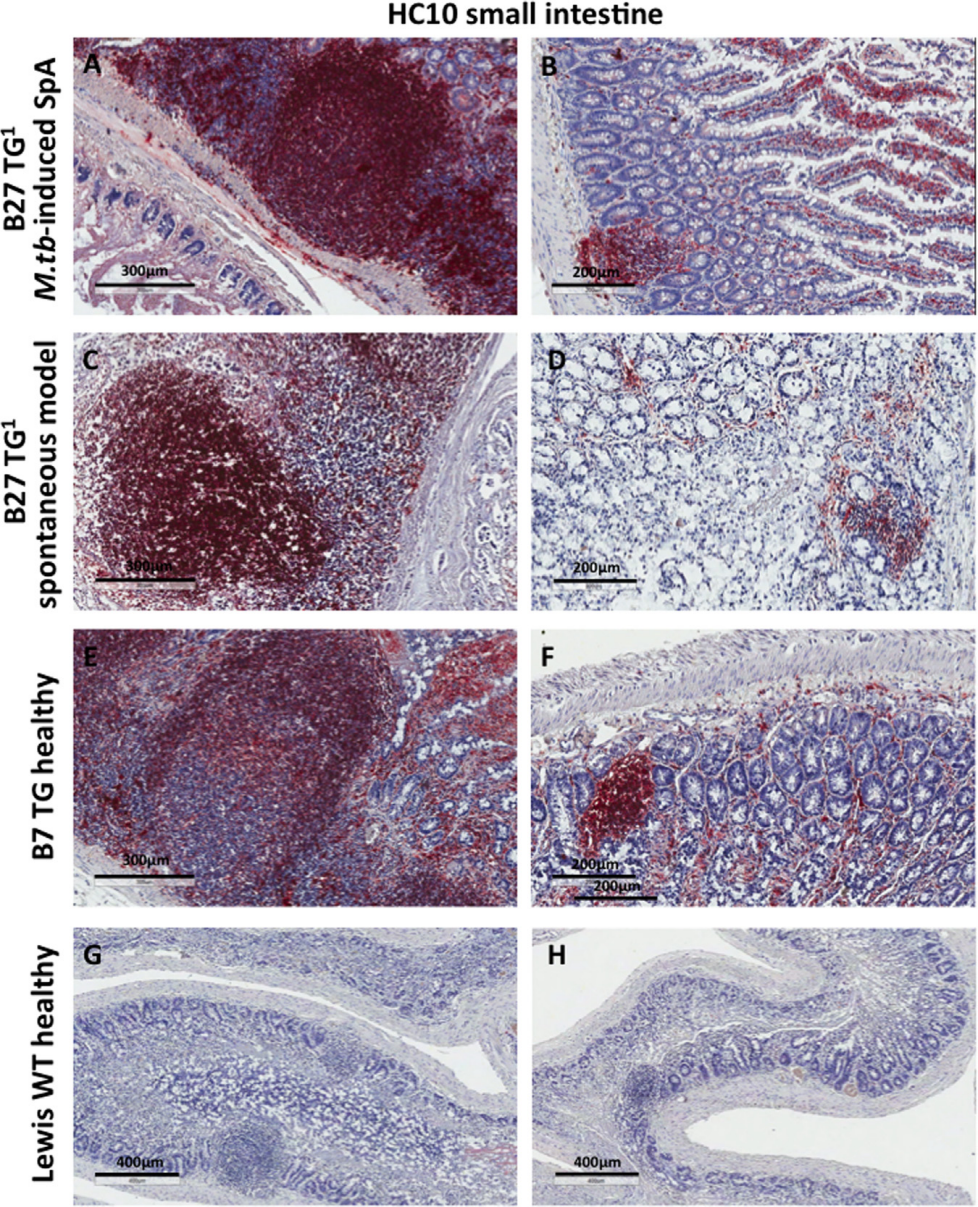
Shows HC10 staining was detectable on mononuclear cells in small intestinal Peyer׳s patches, in lymphoid follicles and in the lamina propria of all transgenic animals. Staining levels were higher for B27 TG^1^ rats with *M.tb*-induced arthritis and spondylitis compared to those without *M.tb* or healthy B7 TG animals.

**Fig. 6:**
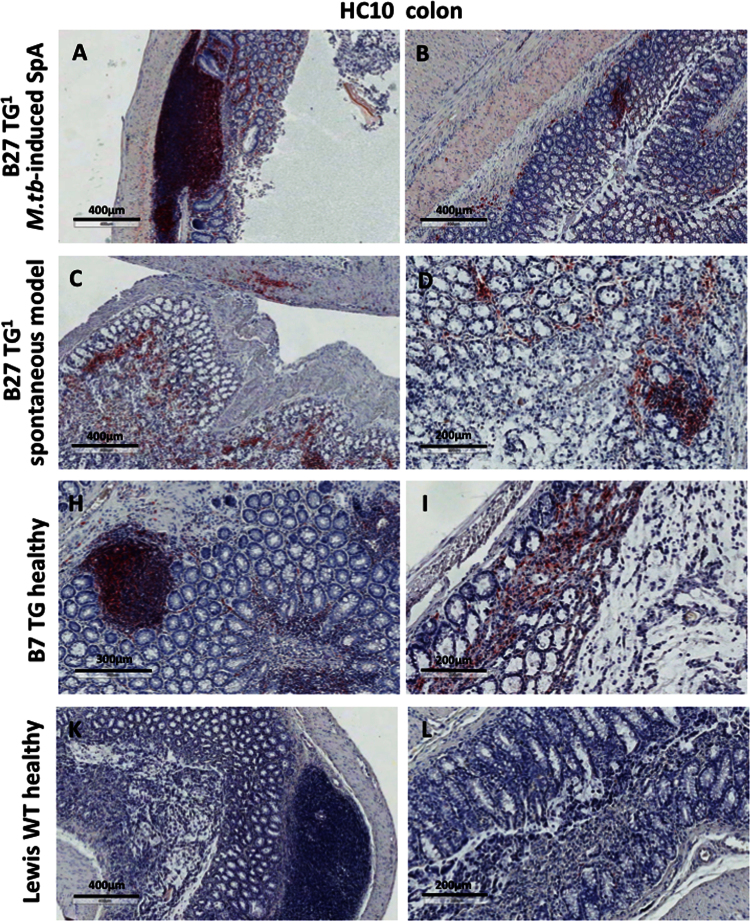
Specific HC10 staining was also seen in the colon.

**Fig. 7:**
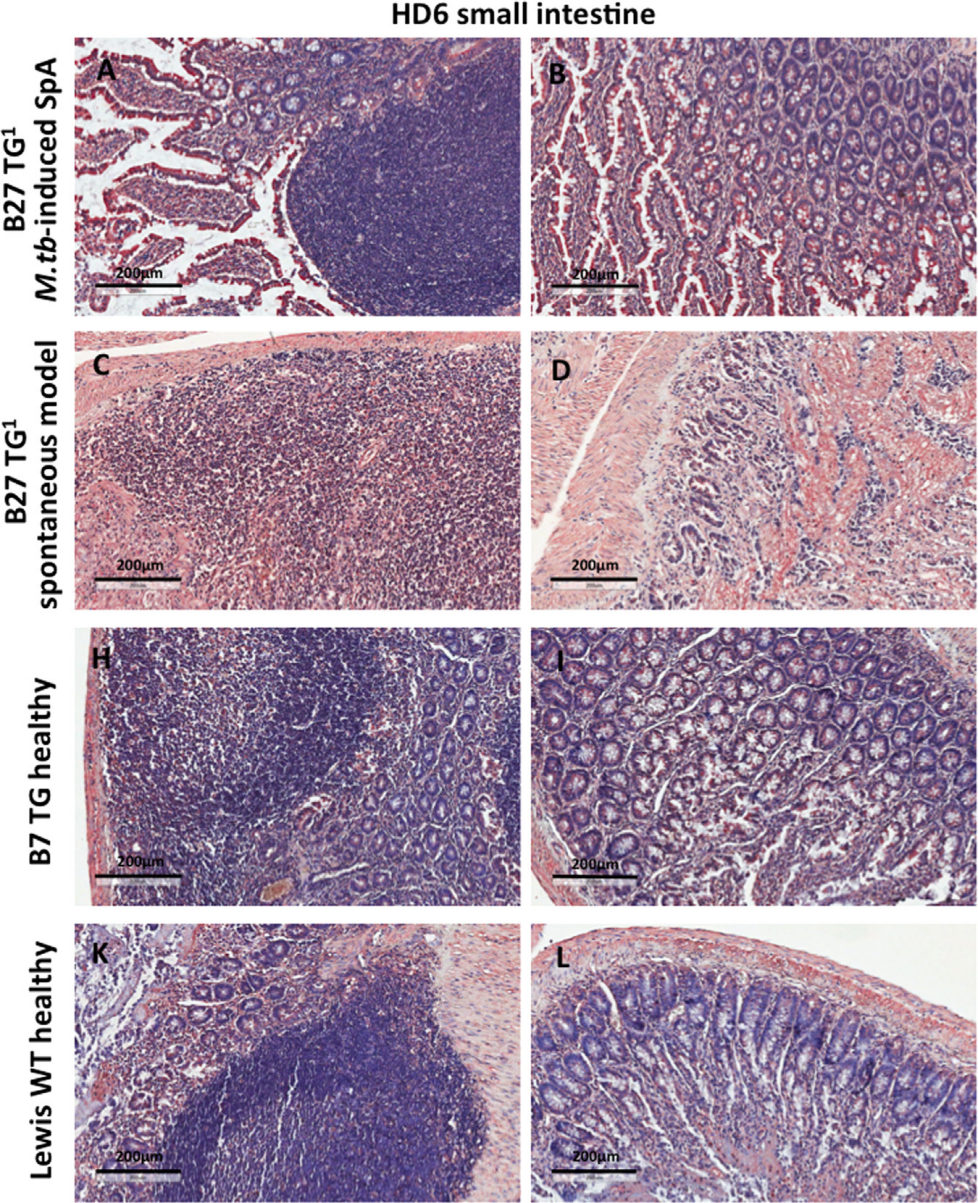
HD6 stained small and large bowel tissues, although with background staining observed (Fig. 7 and data not shown). These data show that NC-B27 are expressed in gut tissue in B27 TG^1^ rats.

**Fig. 8:**
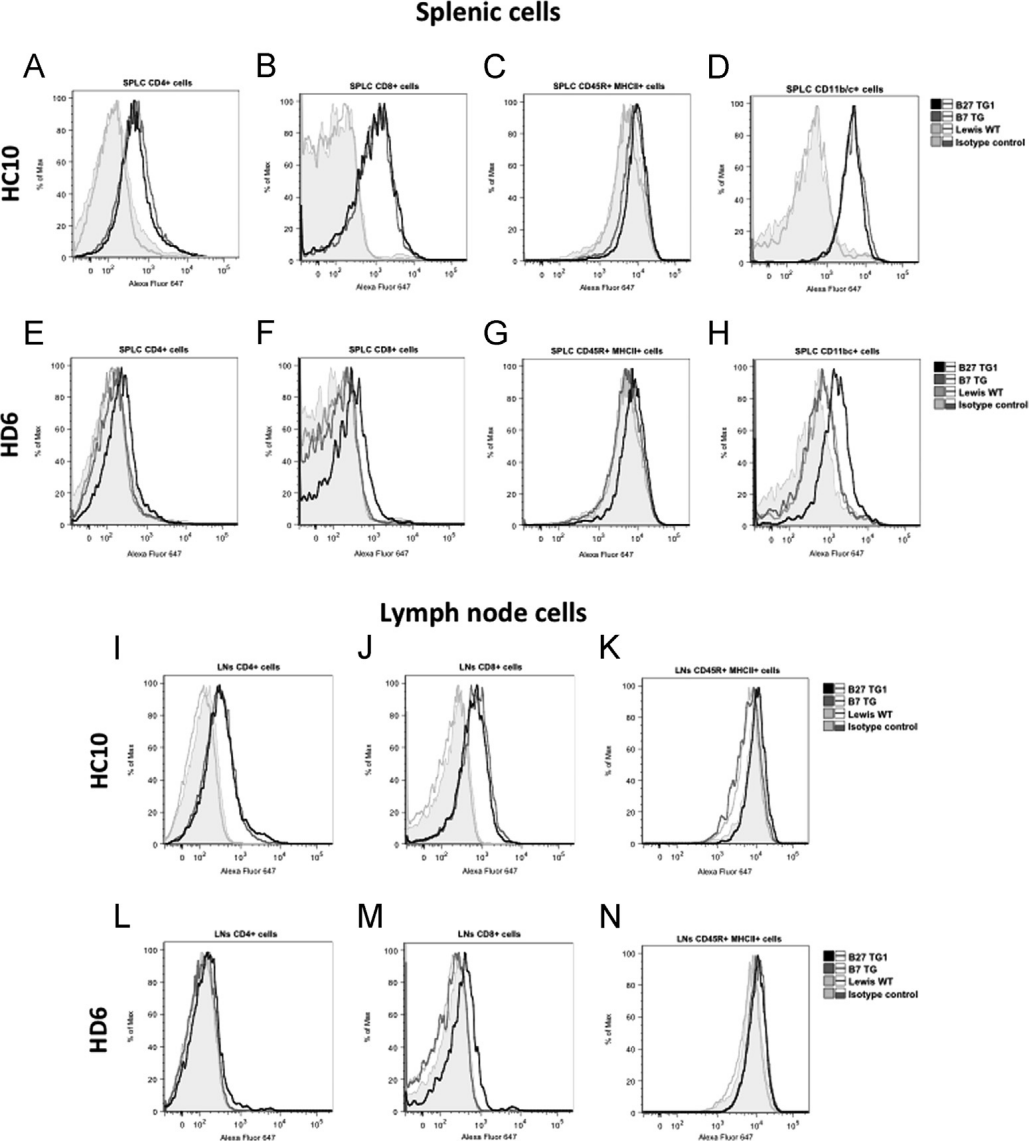
Shows that splenic and lymph node CD45^+^/MHCII^+^ leukocytes from B27 TG^1^ rats with *M.tb*-induced arthritis and spondylitis expressed very low levels of NC-B27 molecules.

**Fig. 9:**
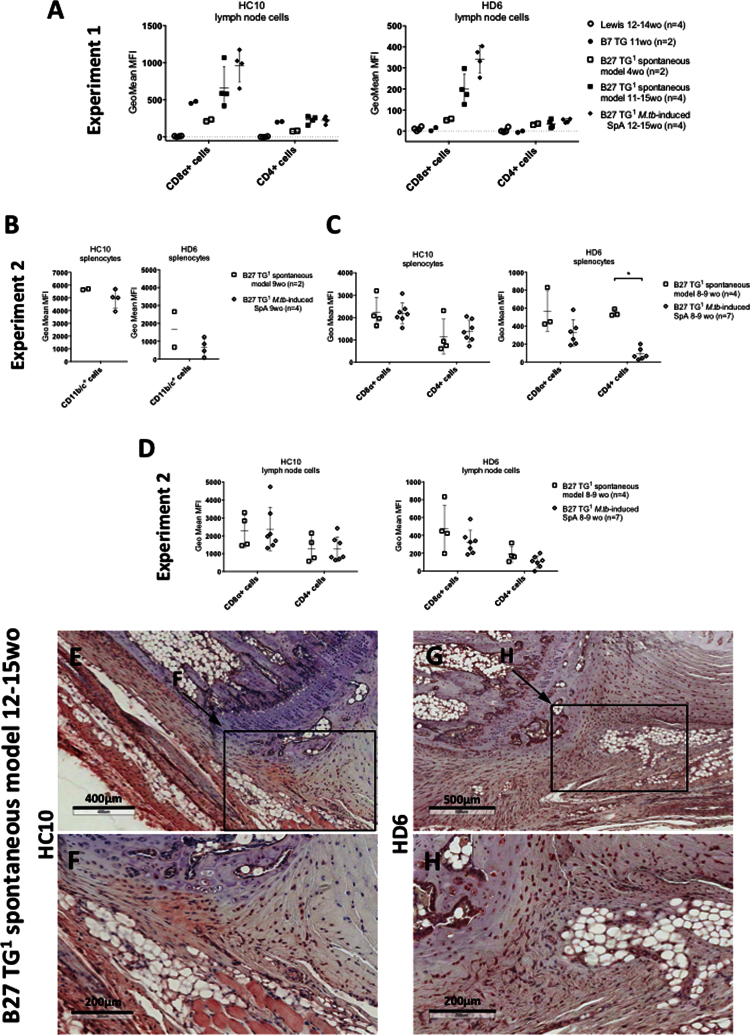
Similar results were observed with cells isolated from B27 TG^1^ lymph nodes +/− *M.tb* (Fig. 9**A**), noting that the CD11b/c^+^ cell population was absent (see Fig. 10**J**). We also investigated HC10 and HD6 staining of splenocytes taken from 8–9 weeks old B27 TG^1^ animals with and without *M.tb*-induced SpA before the appearance of clinical manifestations. HC10 was not significantly altered in splenic CD11b/c^+^, CD8α^+^ or CD4^+^ cells, or on cell populations from LNs (Fig. 9**B–D**). However, we observed an increase in HD6 staining on splenic CD4^+^ cells after *M.tb* treatment (Fig. 9**C** right-hand panel). No HC10 or HD6 staining was observed in splenic and LN cells from age-matched Lewis WT rats (data not shown). Splenic and LN cells from age-matched B7 TG rats stained with HC10, but not HD6, to a similar degree compared with B27 TG^1^ animals (spontaneous model) (Fig. 9**A**).

**Fig. 10:**
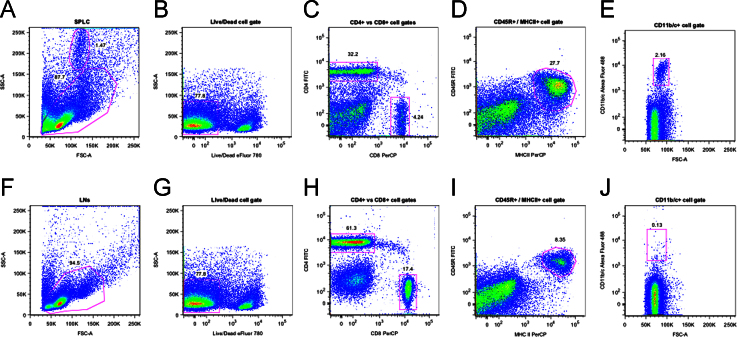
Shows the gating strategy for analysis of spleen and lymph node cell populations studied by FACS.

The immunohistochemistry data show expression of NC-B27 forms (HC10 and HD6 staining) in synovial tissues from B27+ve SpA patients (Fig. 1), and in joint and gastrointestinal tissues from B27 TG^1^ rats with *M.tb*-induced SpA and in healthy WT and B7 TG controls (Fig. 2, Fig. 3, Fig. 4, Fig. 5, Fig. 6, Fig. 7). The flow cytometry data describe and quantify the expression of HC10- and HD6-reactive NC-B27 molecules in spleens and lymph nodes from B27 TG^1^ rats in a spontaneous and *M.tb*-induced SpA before and after disease onset (Fig. 8, Fig. 9, Fig. 10).

## 2 Experimental design, materials and methods

### 2.1 Patients

Human synovial tissue samples were obtained with informed consent and appropriate ethical permission, from B27^+^ SpA patients, including 1 with Ankylosing Spondylitis (AS) fulfilling the New York classification criteria [2], and patients with Rheumatoid Arthritis (RA) fulfilling the EULAR/ACR criteria [3].

### 2.2 Rat-derived cells and tissues

B27 transgenic (TG) rats first generated by Hammer and colleagues spontaneously develop inflammatory gut and joint disease [4]. More recently additional human β2m was introduced, i.e. (21-3×283-2) F_1_ HLA-B27/Huβ2m [5]. We term this model, studied here, as B27 TG^1^. A higher proportion of these B27 TG^1^ male rats spontaneously develop arthritis (~70%, 4–6 months of age) and spondylitis (30–50%, 7–9 months of age) without symptoms of gut inflammation [5], [6], [7]. Early and coordinated onset of these SpA-like disease manifestations can be triggered by immunization with low doses of *M. tuberculosis* (hereafter referred to as “*M.tb*-induced arthritis and spondylitis”) [8], [9]. Splenocytes, lymph node cells (LNs), ankle, tail joints and GI tissues were isolated from B27 TG^1^ rats with spontaneous or induced SpA at age 4–15 weeks. For *M.tb*-induced arthritis and spondylitis [8], 6 week-old B27 TG^1^ rats were immunized with 30–45 μg of heat-inactivated *M.tb* in incomplete Freund׳s adjuvant [8], [9]. (120-4×283-2)F_1_ HLA-B7/Huβ2m TG (B7 TG) and Lewis wild type (WT) animals +/− 200 μg of heat-inactivated *M.tb* in IFA (adjuvant-induced arthritis, AIA model) were used as controls. All animals were bred and housed at the animal facility of the AMC, University of Amsterdam, Netherlands. All animal procedures were carried out in compliance with Institutional Standards for Human Care and Use of Laboratory Animals.

### 2.3 Antibodies

The HC10 antibody stains many or all heavy chain forms (but not beta-2-microglobulin-associated conventional forms) of most human HLA-B and some HLA-A alleles, but does not cross react with rat MHC [10]. HC10 stains HLA-B27 free heavy chains (FHC) including dimers [10], [11]. The HD6 antibody was raised against B27 homodimers using a fully human FAb antibody library (kindly provided by Dynax, MA, USA) as previously described [11], [12], and is more specific for heavy chain forms of HLA-B27. HD6r (same specificity as HD6 but with rat IgG1 Fc region) was used for some stains.

### 2.4 Immunohistochemistry of human and rat tissue samples

Human SpA and RA, and rat paraffin-embedded synovial tissue samples were prepared as previously described [6], [13], [14]. Paraffin-embedded tissue sections were blocked using Peroxidase Blocking Reagent (EnVision™, Dako), than incubated with PBS/1%FBS/10% goat serum and subsequently stained overnight with HC10 or HD6 primary mAb. HC10-stained sections were incubated with HRP-labeled anti-mouse IgG (EnVision™, Dako). HD6-stained sections were incubated with biotinylated goat anti-mouse IgG1 (Southern Biotech) followed by streptavidin-HRP (Dako). Tissue sections were than incubated with AEC^+^ substrate-chromogen (EnVision™, Dako) and counterstained using Mayer׳s hematoxylin. Slides were visualized using an LSM Zeiss confocal microscope, scanned using AperioCS2 Scanner and analyzed using Aperio ImageScope software (Leica Biosystems, UK).

### 2.5 Flow cytometry

Splenocytes and LNs were freshly isolated and immediately stained as described previously [15]. Cells were incubated in blocking buffer, and then stained with primary antibody (HC10, HD6, ME1 or IgG1/IgG2a), followed by incubation with secondary goat anti-mouse antibody (Alexa Fluor 647, Invitrogen). Subsequently, cells were stained for the phenotypic surface markers: CD4 and CD8α or CD45R and MHCII, or CD11b/c. Dead cells were excluded using fixable viability dye eFluor^®^780 (eBioscience). Flow cytometric analysis was performed with BD FACS Canto and data were analyzed using FlowJo Software (TreeStar). Staining was performed in triplicates. Error bars were calculated based on SD mean of the values if 3≥ animals per group. *P* values were determined using nonparametric Mann–Whitney test.

## Funding

OR was supported by Arthritis Research UK, United Kingdom Grant no. 19,611, and by an EMBO travel award. This work was supported by the Oxford National Institute of Health Research (NIHR) Biomedical Research Center, the Oxford NIHR Biomedical Research Unit (PB).
